# Characteristics of offenders referred for psychiatric forensic examination

**DOI:** 10.1192/j.eurpsy.2021.1900

**Published:** 2021-08-13

**Authors:** R. Felhi, G. Hamdi, H. Ben Ammar, R. Ridha

**Affiliations:** Psychiatry, Razi hospital, Manouba, Tunisia

**Keywords:** forensic examination, Clinical characteristics, Criminal reponsibility

## Abstract

**Introduction:**

Forensic psychiatry is facing major challenges related to criminal responsibility with an increasing number of offenses and the entanglement of several factors affecting offenders differently.

**Objectives:**

The aim of this study is to determine the characteristics of offenders referred for forensic psychiatric examination.

**Methods:**

We studied the medical files of all the offenders referred to the forensic psychiatry unit in Razi hospital for an examination between January 2010 and October 2020.

**Results:**

The number of people who have undergone a forensic psychiatric examination was 256. Three files were not usable due to lacking data. The offenders were men in 95.7% (242) of the cases. Their average age was 35 years with a range of 17-53 years. They were mostly single (64%) with primary education (58.1%). Forty percent of the studied population were unemployed and 70% of them lived with their parents. Drug abuse was found in half of the cases and the average number of taken drugs is two illicit substances per person. A criminal record was found in 43% of the cases with an average number of two offenses per person. Offenders were found to suffer from schizophrenia in 29% of the cases, personality disorder in 17% of the cases and from intellectual disability in 16.6% of the cases. No psychiatric disorder was found in 24% of the cases

**Conclusions:**

Despite having in common many vulnerability factors, such as low educational level, unemployment and drug abuse, an important number of offenders referred for forensic psychiatric examination weren’t affected by a psychiatric disorder.

**Disclosure:**

No significant relationships.

